# Outcomes and compliance with standards of care in anti-neutrophil cytoplasmic antibody–associated vasculitis—insights from a large multiregion audit

**DOI:** 10.1093/rap/rky025

**Published:** 2018-07-31

**Authors:** Fiona A Pearce, Catherine McGrath, Ravinder Sandhu, Jon Packham, Richard A Watts, Benjamin Rhodes, Reem Al-Jayyousi, Lorraine Harper, Karen Obrenovic, Peter Lanyon

**Affiliations:** 1 Division of Epidemiology and Public Health, University of Nottingham, Nottingham; 2 Department of Rheumatology, Dudley Group NHS Foundation Trust, Dudley; 3 Institute of Applied Clinical Science, University of Keele, Newcastle-under-Lyme; 4 Department of Rheumatology, Ipswich Hospital, Ipswich; 5 Norwich Medical School, University of East Anglia, Norwich; 6 Department of Rheumatology, University Hospitals Birmingham NHS Foundation Trust, Birmingham; 7 Department of Nephrology, University Hospitals of Leicester NHS Trust, Leicester; 8 Institute of Clinical Sciences, University of Birmingham, Birmingham; 9 Clinical Audit Department, Dudley Group NHS Foundation Trust, Dudley; 10 Department of Rheumatology, Nottingham University Hospitals NHS Trust; 11 Department of Rheumatology, Nottingham NHS Treatment Centre, Nottingham, UK

**Keywords:** ANCA-associated vasculitis, audit, cyclophosphamide, routine care, survival

## Abstract

**Objectives:**

We aimed to conduct a large audit of routine care for patients with ANCA-associated vasculitis.

**Methods:**

We invited all 34 hospitals within one health region in England to undertake a retrospective case note audit of all patients newly diagnosed or treated with CYC or rituximab (RTX) for ANCA-associated vasculitis from April 2013 to December 2014. We compared clinical practice to the British Society for Rheumatology guidelines for the management of adults with ANCA-associated vasculitis and the use of RTX with the National Health Service (NHS) England commissioning policy and National Institute for Health and Care Excellence (NICE) technology appraisal.

**Results:**

We received data from 213 patients. Among 130 newly diagnosed patients, delay from admission to diagnosis ranged from 0 to 53 days (median 6, interquartile range 3–10.5) for those diagnosed as inpatients. BVAS was recorded in 8% of patients at diagnosis. Remission at 6 months was achieved in 83% of patients. The 1-year survival was 91.5%. A total of 130 patients received CYC for new diagnosis or relapse. The correct dose of i.v. CYC (within 100 mg of the target dose calculated for age, weight and creatinine) was administered in 58% of patients. A total of 25% of patients had an infection requiring hospital admission during or within 6 months of completing their CYC therapy. Seventy-six patients received RTX for new diagnosis or relapse. A total of 97% of patients met the NHS England or NICE eligibility criteria. *Pneumocystis jiroveci* pneumonia prophylaxis (recommended in the summary of product characteristics) was given in only 65% of patients.

**Conclusion:**

We identified opportunities to improve care, including compliance with safety standards for delivery of CYC. Development of a national treatment protocol/checklist to reduce this heterogeneity in care should be considered as a priority.

**Key messages**

- Infections requiring hospital admission occurred in 25% of ANCA-associated vasculitis patients receiving cyclophosphamide.
- Only 58% of ANCA-associated vasculitis patients on i.v. cyclophosphamide received the correct dose (within a 10% tolerance).
- Tertiary referral centres treated ANCA-associated vasculitis sooner and more patients received correct doses of cyclophosphamide.

## Introduction

ANCA-associated vasculitis (AAV) has a high mortality, with the greatest mortality risk occurring within the first year after diagnosis. Yet there is very little data on the process and outcomes of routine National Health Service (NHS) clinical care during this time period, aside from individual centre case series or small clinical trials. However, the patient charity Vasculitis UK frequently states that its members report variations in clinical practice and outcomes throughout the UK.

Remission induction of AAV with CYC is probably the most frequent non-cancer indication for cytotoxic chemotherapy. National guidance from the National Chemotherapy Advisory Group, designed to ensure the quality and safety of all chemotherapy services, is also applicable to non-cancer chemotherapy [1]. The publication of British Society for Rheumatology (BSR) guidelines on the management of adults with AAV [2] and an NHS England commissioning policy for the use of rituximab (RTX) in AAV [3], followed by a National Institute for Health and Care Excellence (NICE) technology appraisal [4], provided further benchmarks against which to assess care.

The aim of this audit was to compare current practices, compliance with national guidelines and outcomes within a large, representative and geographically defined area in England.

## Methods

Rheumatology units in all 34 hospitals within one of the four health regions in England (Midlands and East, population 6 980 000) were invited to undertake a retrospective case note audit of all AAV patients who were either newly diagnosed or treated with CYC or RTX for relapse from April 2013 to December 2014. Each invitation recommended involving the hospital’s nephrology unit. Patients were considered to have AAV if this was their diagnosis given by a hospital physician.

We developed and piloted a set of audit questions derived from the BSR guidelines, NHS England and NICE technology appraisal. We provided guidance on how to identify cases through departmental database, clinic letter, day-case and inpatient admissions searches. Data were collected locally and uploaded onto a web-based survey. Survey software was compliant with International Organization for Standardization 27001, the internationally recognized gold standard for information security systems, hosted by the Dudley Group NHS Foundation Trust Clinical Audit Department. The form ensured complete data entry for most questions, as it could not be submitted unless questions were answered. Data were collected on the specialty of the attending physician, place of diagnosis, date of symptom onset, admission or first clinic appointment, diagnosis, BVAS organ systems involved, details of remission induction, documentation of disease activity and damage, compliance with CYC and RTX safety standards and outcomes, including hospitalization for infection and death. Diagnostic delay was retrospectively estimated from information recorded in the medical notes and was defined as the time from the date of the first reported symptom attributed to AAV to the date of diagnosis. Patient age, sex, subtype of AAV diagnosis and ANCA type were collected later, after completion of initial data entry.

Tertiary referral centres were defined as hospitals that at least two other hospitals reported making tertiary referrals to for AAV. Tertiary referral centres were compared with the other non-tertiary centres using the chi-squared test for categorical data and Wilcoxon’s rank sum test for continuous non-normally distributed data. The 1-year survival was calculated using the Kaplan–Meier method. The odds ratio (OR) for infection was estimated using logistic regression, and the hazard ratio (HR) for death was estimated using Cox regression; both were adjusted for confounders (age and renal involvement). Available case analysis was used where there were missing data. Statistical analyses were performed using Stata 14 statistical software (StataCorp, College Station, TX, USA). This project was approved by the audit department of each trust that participated.

## Results

We received data about 213 patients from 20 units: 130 newly diagnosed patients and an additional 83 relapsing patients who were treated with CYC or RTX during the audit period. In each unit, 1–41 patients were included, with 144 (68%) treated primarily by rheumatology and 69 (32%) treated by nephrology.

There were no missing data for audit outcomes. For the data collected at a later stage there were some missing data [age (1%), sex (7%), diagnosis (10%) and ANCA type (6%)].

### New diagnosis

Baseline characteristics of the 130 newly diagnosed patients are shown in Table 1. The median age was 67 years [interquartile range (IQR) 56–73], 52 patients (43%) were female, 57 (49%) had granulomatosis with polyangiitis, 49 (42%) had microscopic polyangiitis, 10 (9%) had eosinophilic granulomatosis with polyangiitis, 72 (55%) were diagnosed as inpatients and 58 (45%) were outpatients. The frequency of organ involvement at diagnosis is shown in Table 2. The diagnostic delay from first symptoms to diagnosis was a median of 2.6 months (IQR 1.2–6.1). It was shorter in those diagnosed as inpatients [1.8 (95% CI 0.9, 3.7)] compared with outpatients [4.1 (95% CI 2.0, 12.6)]. Among inpatients, the delay from admission to diagnosis ranged from 0 to 53 days (median 6, IQR 3–10.5). The BVAS was recorded in 10/130 patients (8%) at diagnosis and 8/121 (7%) at 6 months. The first choice of agent for remission induction was CYC in 99 patients (76%), RTX in 6 (5%) and other agents in 25 (19%). The prednisolone dose at treatment initiation was a median of 55 mg (IQR 40–60, range 0–100) and additional i.v. methylprednisolone was administered in 60 patients (46%). At 6 months the prednisolone dose was a median of 9.5 mg (IQR 5–10, range 0–60) among the patients documented to be in remission. Remission at 6 months was achieved in 101 patients (83%). The 1-year survival was 90.8%. In the 76 patients who were recorded as having renal involvement, 1-year survival was 85.5%.

**Table 1:** Newly diagnosed patients (*n*=130)

Characteristics	Values
Age, median (IQR), years	67 (56–73)
Female	52 (43)
Male	68 (57)
GPA	57 (49)
MPA	49 (42)
EGPA	10 (9)
PR3-ANCA	52 (43)
MPO-ANCA	55 (45)
ANCA negative	9 (8)
p-ANCA only (not PR3/MPO)	3 (3)
c-ANCA only (not PR3/MPO)	1 (1)
BVAS organ system involved at diagnosis	
Constitutional symptoms	88 (73)
Renal	76 (63)
Chest	62 (50)
ENT	55 (47)
Cutaneous	30 (25)
Nervous system	28 (23)
Mucous membranes/eyes	20 (17)
Abdominal	13 (11)
Cardiovascular	8 (7)
Audit outcomes	
Delay from first symptom to diagnosis, median (IQR), months	2.6 (1.2–6.1)
BVAS recorded	
At diagnosis	10 (8)
At 6 months	8 (7)
First choice of remission induction treatment	
CYC	99 (76)
RTX	6 (5)
Other agent	25 (19)
Glucocorticoids	
Prednisolone at diagnosis, median (IQR), mg	55 (40–60)
Additional i.v. methylprednisolone	60 (46%)
Prednisolone at 6 months, median (IQR), mg	10 (5–10)
Remission at 6 months	101 (83)
Survival at 1 year	
All patients (*n* = 130)	90.8% (95% CI 84.3, 94.7)
Patients with documented renal involvement (*n* = 76)	85.5% (95% CI 75.4, 91.7)

Values are *n* (%) unless stated otherwise. c-ANCA: cytoplasmic ANCA; EGPA: eosinophilic granulomatosis with polyangiitis; GPA: granulomatosis with polyangiitis; MPA: microscopic polyangiitis; MPO: myeloperoxidase; PR3: proteinase 3; p-ANCA: perinuclear ANCA.

**Table 2:** Risk of infection and death in newly diagnosed patients treated with oral compared with i.v. CYC

CYC route	Infection			Mortality		
	*n* (%)	Crude OR for infection	Adjusted OR^a^	*n* (%)	Crude HR for death	Adjusted HR^a^
I.v.	15/74 (20.2)	1	1	9/74 (12.2)	1	1
PO	9/25 (36.0)	2.2 (0.8–6.0)	1.8 (0.6–5.1)	6/25 (24.0)	2.3 (0.8–6.5)	1.7 (0.5–5.3)

a Adjusted for age and renal involvement.

Of the 99 newly diagnosed patients treated with CYC, 74 (75%) received i.v. doses and 25 (25%) oral. A total of 24 patients (24%) had infections requiring hospitalization during or within 6 months of CYC treatment. The 1-year survival in this subgroup was 87.9%. Compared with i.v. doses, the crude OR for infection with oral CYC was 2.2 (95% CI 0.8, 6.0) and the HR for death was 2.3 (95% CI 0.8, 6.6). Once adjusted for age and renal involvement, the OR for infection remained elevated at 1.8 (95% CI 0.6, 5.1) and the HR for death was 1.7 (95% CI 0.5, 5.3) (Table 2).

### CYC safety standards 

A total of 130 patients received CYC for new diagnosis or relapse: 101 (78%) received i.v. doses and 29 (22%) received oral doses (Table 3). The correct dose of i.v. CYC could be calculated for 95 (94%) of these patients based on BSR recommendations for age, weight and renal function, within a tolerance of ±100 mg, based on the dose banding for cancer chemotherapy introduced by NHS England, which uses dose bands within 5–10% of the target dose [5]. The correct dose was administered in only 50 patients (58%), with underdosing in 32 (34%) and overdosing in 13 (8%). The most common dose given to those who received an unrecommended dose was 1000 mg, and those who were underdosed were on average younger and heavier and those who were overdosed were on average older and lighter than the whole cohort. At least one full blood count was checked 7–10 days after the first dose of oral or i.v. CYC in 119 patients (92%). The total cumulative CYC dose per patient was a median of 6 g (IQR 4–9, range 0.1–21). No patients exceeded a lifetime exposure of 25 g. Mesna was given in 99 patients (76%). *Pneumocystis jiroveci* pneumonia (PJP) prophylaxis was given in 106 patients (82%). Thirty-three patients (25%) had an infection requiring hospital admission during or within 6 months of completing their CYC therapy.

**Table 3:** Patients treated with CYC for new diagnosis or relapse (*n* = 130)

Characteristics	Values
Age, median (IQR), years	65 (56–72)
Female	50 (42)
Male	68 (58)
GPA	64 (58)
MPA	39 (35)
EGPA	8 (7)
PR3-ANCA	58 (49)
MPO-ANCA	46 (39)
ANCA negative	8 (7)
p-ANCA only (not PR3/MPO)	4 (3)
c-ANCA only (not PR3/MPO)	2 (2)
Treatment	
Oral CYC	29 (22)
I.v. CYC	101 (78)
Audit outcome	
Correct dose of i.v. CYC, within 100 mg	50 (58)
Underdosed >100 mg	32 (34)
Overdosed >100 mg	13 (8)
FBC was checked 7–10 days after the first dose	119 (92)
Total cumulative dose of CYC, median (IQR) [range], g	6 (4–9) [0.1–21]
Co-prescription of Mesna	99 (76)
Co-prescription of PJP prophylaxis	106 (82)
Admission with infection during or within 6 months of CYC therapy	33 (25)

All values are *n* (%) unless stated otherwise. c-ANCA: cytoplasmic ANCA; EGPA: eosinophilic granulomatosis with polyangiitis; FBC: full blood count; GPA: granulomatosis with polyangiitis; MPA: microscopic polyangiitis; MPO: myeloperoxidase; PR3: proteinase 3; p-ANCA: perinuclear ANCA.

### RTX safety standards and compliance with NICE/NHS criteria for eligibility

A total of 76 patients received RTX for new diagnosis or relapse (Table 4). The dosing schedule among the 16 patients newly diagnosed was 1 g × 2 in 15 patients (94%) and 375 mg/m^2^ × 4 in 1 patient (6%). The dosing schedule among the 60 patients treated for relapse was 1 g × 2 in 35 patients (58%), 375 mg/m^2^ × 4 in 16 (27%) and 1 g × 1 in 7 (12%). A total of 74 patients (97%) met NHS England or NICE eligibility criteria. PJP prophylaxis (recommended in the summary of product characteristics) was given in only 49 patients (65%). Igs were checked prior to RTX in 63/68 patients (93%).

**Table 4:** Patients treated with RTX for new diagnosis or relapse (*n* = 76)

Characteristics	Values
Age, median (IQR), years	50 (36–63)
Female	34 (47)
Male	39 (53)
GPA	60 (82)
MPA	11 (15)
EGPA	2 (3)
PR3-ANCA	60 (82)
MPO-ANCA	11 (15)
ANCA negative	1 (1)
p-ANCA only (not PR3/MPO)	1 (1)
c-ANCA only (not PR3/MPO)	0
Treatment	
RTX given for new diagnosis^a^	16 (21)
Regimen	
1 g × 2	15 (94)
375 mg/m^2^ × 4	1 (6)
Diagnosis, %	
GPA	67
MPA	33
EGPA	0
RTX given for relapse	60 (79)
Regimen	
1 g × 2	35 (58)
375 mg/m^2^ × 4	16 (27)
1 g × 1	7 (12)
Diagnosis	
GPA	95
MPA	2
EGPA	2
Audit outcomes	
Treated at referral centres	48 (63)
Treated at other centres	28 (37)
Igs checked prior to treatment	63/68 (93)
Co-prescription of PJP prophylaxis	49 (65)
Met NICE technology appraisal/NHS England eligibility criteria	74 (96)

All values are *n* (%) unless stated otherwise.

a First choice treatment (*n* = 6), after switching (*n* = 10).

c-ANCA: cytoplasmic ANCA; EGPA: eosinophilic granulomatosis with polyangiitis; GPA: granulomatosis with polyangiitis; MPA: microscopic polyangiitis; MPO: myeloperoxidase; PR3: proteinase 3; p-ANCA: perinuclear ANCA.

### Tertiary and non-tertiary referral centres

Of the 130 newly diagnosed patients, 45 (35%) were treated in four tertiary referral centres and 85 (65%) were treated in 16 non-tertiary centres (Table 5). The delay between admission and diagnosis was a median of 4 days (IQR 2–13) in tertiary referral centres compared with 7 (IQR 4–11) in non-tertiary centres (*P* = 0.4). The delay between diagnosis and starting immunosuppression was a median of 4 days (IQR 3–10) in tertiary referral centres and 9 (IQR 3–19) in non-tertiary centres (*P* = 0.01). Of the 130 patients treated with CYC, 42 (32%) were treated in tertiary referral centres and 88 (68%) were treated in non-tertiary centres. The correct dose of i.v. CYC was given in 23/32 patients (72%) treated in tertiary referral centres compared with 29/57 patients (51%) treated in non-tertiary centres (*P* = 0.05). Infections requiring hospital admission occurred in 9 patients (21%) treated in tertiary referral centres compared with 24 patients (27%) treated in non-tertiary centres (*P* = 0.5). Patients who received RTX were more commonly treated in tertiary referral centres [48 (63%) compared with 28 (37%) treated in non-tertiary centres]. PJP prophylaxis was prescribed to 73% of patients on RTX treated in tertiary referral centres compared with 52% treated at non-tertiary centres (*P* = 0.07).

**Table 5:** Comparison of referral and non-referral centres

	Referral centres	Non-referral centres	*P*-value
Newly diagnosed patients, *n* (%)	45 (35)	84 (65)	
Delay between admission and diagnosis, median (IQR), days	4 (2–13)	7 (4–11)	0.4
Delay between diagnosis and starting immunosuppression, median (IQR), days	4 (3–10)	9 (3–19)	0.01
Patients treated with CYC, *n* (%)	42 (32)	88 (68)	
Correct dose of i.v. CYC	23/32 (72)	29/57 (51)	0.05
Admission with infection during or within 6 months of CYC therapy, *n* (%)	9 (21)	24 (27)	0.5
Patients treated with RTX, *n* (%)	48 (63)	28 (37)	
Co-prescription of PJP prophylaxis, %	73	52	0.07

## Discussion

### Main findings

We identified a cohort of 213 patients receiving routine clinical care for AAV in a large health region of England. We found long delays between admission and diagnosis in some inpatients diagnosed with AAV (maximum >7 weeks). We found that a guideline-recommended dose of i.v. CYC, based on age, weight and renal function, was prescribed in <60% of patients and that adherence to other safety standards for monitoring and prophylactic medication had room for improvement. RTX treatment was prescribed to eligible patients in compliance with the NICE technology appraisal and NHS England commissioning policy, but there was opportunity to improve the number of patients co-prescribed PJP prophylaxis as is recommended in the summary of product characteristics. We found that the delay between diagnosis and starting immunosuppressive treatment and prescription of the correct dose of CYC were significantly better in tertiary referral centres, which provided care for much larger numbers of patients, than non-tertiary centres.

We found that 25% of patients on CYC were admitted with infection during or in the 6 months after CYC treatment, which is higher than expected based on previous studies [6–8]. Our best estimates of the effect of giving CYC orally compared with the i.v. route, once adjusted for the effects of age and renal involvement, were that it increased infections by 80% and the risk of death by 70%, but our CIs were wide, as our sample size is small, and these were not statistically significant.

### Strengths and limitations

This is the largest collaborative audit of a rare autoimmune rheumatic disease and the first time services for people with AAV have been able to benchmark their care not just against standards, but also against other providers. Its main strength is in the large and unselected group of patients who were diagnosed with AAV by their rheumatologist or renal physician, across a range of different-sized health care providers ranging from smaller district general hospitals to large tertiary referral centres. It therefore enables a representative overview of the process and outcome of care in England that cannot be adequately gained from existing clinical trial reports or cohorts from single centres.

As it is an audit, there are limitations, particularly incomplete case capture. Cases were contributed by 20 (59%) of 34 invited units. A cohort of 130 newly diagnosed adult patients were identified over 21 months in an adult catchment population of 6 980 000, which equates to an incidence rate of 10.6 per million person-years, suggesting we identified ∼50% of all expected incident cases [9], which compares favourably with the first year of the national rheumatoid and inflammatory arthritis audit, which captured ∼42% of expected cases [10]. The demographics of cases included in the audit were very similar to a recent epidemiological study [11] and were received from the expected mix of district general and tertiary referral hospitals. Data were collected and entered by a large team of people, which may lead to variations in interpretation of the questions, however, a set of explanatory notes covering each question minimized this risk (see supplementary data, AAV Audit 2015 Guidance notes for completion, available at *Rheumatology Advances in Practice* online). There are some missing data, particularly for baseline demographics, as these were collected later, however, the main outcomes have no missing data due to the electronic form not allowing submission until all questions were answered.

The audit captured data from patients treated between April 2013 and December 2014, while the BSR, NHS England and NICE standards were introduced during this period, although CYC dosing guidance is identical to the 2007 guideline. This might explain the low use of BVAS to document outcomes (which these documents recommend/require). BVAS training can be completed online [12] and the BVAS takes <3 min to complete [13]. It is possible that care has improved post-audit in response to these guidelines.

### Real-life audit comparisons to clinical trials

Our audit is the first description of routine care of patients with AAV and compliance with safety standards across a large health region. The baseline characteristics of included patients are similar to other UK epidemiological studies from the Midlands and Norfolk [11, 14]. Our cohort also had remission (83%) [6] and survival rates (1-year survival 90.8%) similar to other studies [8, 15–17].

Of note, our audit of routine practice found higher rates of serious infection than in the European Vasculitis Study Group clinical trials [18]. This is likely to be influenced by the selected populations, and possibly by the protocolized treatment, in clinical trials. For example, the CYCLOPS trial (Randomised trial of daily oral versus pulse Cyclophosphamide as therapy for ANCA-associated Systemic Vasculitis) reported 11% of patients had severe infections requiring hospital treatment during a median of 18 months follow-up, so the follow-up time was longer than in our audit, and the trial excluded those with creatinine >500 μmol/l and age <18 or >80 years, which are risk factors for serious infection [6]. Based on these exclusion criteria alone, 7% of the patients included in our audit would have been excluded from the CYCLOPS trial. Our results are similar to a recent study of all patients presenting to a single centre, where 22% of patients receiving CYC for AAV were admitted with infection during the first year after commencing treatment [19]. This suggests 22–25% may be a realistic estimate of the risk of hospitalisation with infection in the year after starting CYC and patients should receive counselling about pre-treatment.

We did not find a statistically significant increased risk of infection or death in patients treated with oral compared with i.v. CYC, however, our audit was underpowered for this analysis. A previous meta-analysis of three randomized controlled trials comparing oral *vs* i.v. CYC found i.v. CYC conferred a significantly lower risk of infection [OR 0.45 (95% CI 0.23, 0.89)] [20].

Our findings reflect some of the findings common to rare diseases, which are highlighted in the UK Strategy for Rare Diseases [21]. For example, people with rare diseases are often slow to benefit from advances in treatment [21], and it is notable that although the minority of patients (41%) overall were treated at tertiary referral centres, the majority of patients receiving RTX (63%) were treated in tertiary referral centres. Although tertiary referral centres are more likely to treat severe disease, they may also be quicker to embrace new treatments, facilitated by these centres fulfilling the ‘specialised centre’ requirements of the NHS England policy for access to this drug.

### Clinical implications and conclusion

We identified opportunities to improve our care, including improving compliance with safety standards for the delivery of CYC for the 42% of patients who received an incorrect dose of i.v. CYC. Our comparison of oral *vs* i.v. CYC adds to the level of certainty from other studies that oral CYC has greater toxicity. Development of a national treatment protocol/checklist to reduce heterogeneity in care should be considered as a priority. More than half of patients were diagnosed as inpatients, and we found a long delay between admission and diagnosis in some patients (up to 53 days). Increased awareness among acute admitting physicians and earlier ANCA testing could reduce diagnostic delay and perhaps reduce organ damage among newly diagnosed patients. Our finding that 25% of patients on CYC were admitted with infection during or following CYC therapy is higher than in clinical trials and requires increased vigilance for infection and changes to the expectations we give patients when counselling them before starting treatment.

## Supplementary Material

Supplementary Data
