# Supplementary material for: Outcomes and compliance with standards of care in anti-neutrophil cytoplasmic antibody–associated vasculitis—insights from a large multiregion audit
Source: Rheumatol Adv Pract. 2018 Jul 31;2(2):rky025. doi: 10.1093/rap/rky025 (PMC6649985; doi:10.1093/rap/rky025)
Supplement: Supplementary Data [file rky025_supp.docx]

*1*

**ANCA Vasculitis Audit Guidance Notes November 2015**

***ANCA Associated Vasculitis (AAV) Audit 2015
Guidance notes for completion***

*Please enter data via* [*http://dudleygroup.nhs.uk/regional-anca-associated-vasculitis-aav-audit/*](http://dudleygroup.nhs.uk/regional-anca-associated-vasculitis-aav-audit/)

*Password:*

*The form has buttons at the bottom to Reset, Save and move onto the Next page.*

*If you make a mistake, press the ‘Reset’ button, which resets the current page only.*

*If you need to save the form, press the ‘Save’ button – and follow the instructions on how to return later.*

*When you complete the form and submit it, then the data are emailed (securely) to the audit department in*

*Dudley. There are no data stored on the website.*

*Each question in this audit has been derived from either the BSR Guidelines, NICE guidance or NHS England Commissioning Policy, and is considered self-explanatory unless a specific guidance note has been provided below. However, please contact Dr Fiona Pearce (*[*fiona.pearce2@nhs.net*](mailto:fiona.pearce2@nhs.net)*), lead registrar in the East Midlands, if you have any queries.*

2

ANCA Vasculitis Audit Guidance Notes November 2015

***UNIT DATA***

*This should be completed by either the Head of Service or delegated e.g. to the Vasculitis Lead.*

***Q1: How many new and follow up AAV patients have been seen in your department between the 1 April 2013 and 31 December 2014?***

*There are several methods to obtain this data, each of which have performed well at pilot sites*

1. *Search departmental database and/or date-limited search of departmental clinic letters for patients with a diagnosis of ANCA-associated vasculitis and any of its subtypes; Granulomatosis with Polyangiitis (Wegener’s), Microscopic Polyangiitis, and Eosinophilic Granulomatosis with Polyangiitis (Churg-Strauss syndrome). It may also be worth searching for MPO vasculitis or PR3 vasculitis. Please note that* ***ANCA negative*** *patients who have one of these diagnoses* ***should be included****; but please* ***do not include*** *any other types of systemic vasculitis.*
2. *Search daycase diary or Hospital Episode Statistics (HES) via your business analyst (ICD-10 codes M31.3 Wegener’s granulomatosis, M30.1 Churg Strauss, M31.7 Microscopic Polyangiitis) for activity in the audit time period. This will only identify patients who have received IV day case treatment or who have been admitted for any reason.*
3. *Estimation based on recall of cases by colleagues in the department. Please record on the proforma which method was used to obtain the estimate.* ***Do you use a local / regional Cyclophosphamide protocol?***

*If this is used, does it contain a standard GP information letter about cyclophosphamide, management of the likely side effects and who to contact if problems arise, as recommended by 'Chemotherapy Services in England: Ensuring quality and safety. A report from the National Chemotherapy Advisory Group, 2009'*

***Q2: ANCA testing. What is the standard turnaround time in your local lab?***

*The BSR guideline suggests audit topics, including “Availability of ANCA results within 1 working day”.*

*If you are unsure, please contact your immunology laboratory who will have a timetable of when tests are run, and a policy on standard and urgent reporting.*

***Q3: Fertility preservation***

*The BSR guideline recommends that “Patients should be counselled about the possibility of infertility following CYC treatment and offered fertility preservation.”*

*This question asks what services are routinely available for this situation in your centre.*

***Q4-5: Referrals, Birmingham Vasculitis Activity Score (BVAS) training, and Research***

*These questions relate to the NHS England Commissioning Policy criteria for use of Rituximab in AAV, which includes.*

* The decision regarding rituximab maintenance has been made at, or in conjunction with, a specialised centre AND*

* The person has been provided with the opportunity to be considered for any suitable clinical trials AND  The person is registered on the UKIVAS database, to enable identification of use and outcome of treatment.*

*3*

**ANCA Vasculitis Audit Guidance Notes November 2015**

***INDIVIDUAL DATA FOR ALL PATIENTS NEWLY DIAGNOSED 1st APRIL 13 – 31st DECEMBER 14***

*The BSR guideline states that “The essential principles of management are*

1. *Rapid diagnosis.*
2. *Rapid initiation of treatment.*
3. *Early induction of remission to prevent organ damage.*
4. *Maintenance of remission with the aim of eventual drug withdrawal.*
5. *Prevention of drug toxicity.”*

*You only need to complete either* ***Section A (inpatient)*** *or* ***Section B (outpatient)*** *depending on which setting the diagnosis was made in. The questions in this section all relate to the above management principles and are not numbered. Please record in the calendar box the approximate date of onset of the patient’s symptoms, the date of diagnosis, and the date treatment was commenced. Please give specific dates if possible (either accurate or approximate). If you know the month but not the day please enter the 1st of the month.*

***Formal documentation in patient record of disease activity, damage, and prednisolone dose.***

***At Diagnosis***

1. ***BVAS score***

*The BSR guideline recommends “A validated tool should be used to assess disease activity and extent of disease”. If this is not recorded, please leave blank and proceed to the next question.*

1. ***Asssessment of disease activity and extent***

*As the Birmingham Vasculitis Activity Score (BVAS) is the most commonly used tool, the 9 BVAS organ systems have been included. Please answer this section by ticking whether each system was specifically documented as being either affected, not affected, or whether there was no documentation that this system had been assessed. If you wish to become trained and certified in Birmingham Vasculitis Activity Score and vasculitis damage index (BVAS & VDI) this is available at https://bvasvdi.org/*

1. ***Initial oral prednisolone dose***

*The BSR guideline recommends “Induction therapy for AAV includes treatment with high dose GCs in combination with another immunosuppressive agent...” “GCs are usually given as daily oral prednisolone, initially at relatively high doses (1 mg/kg up to 60 mg), with the dose rapidly reduced to 15 mg prednisolone at 12 weeks.”*

*If oral prednisolone was prescribed, please enter the initial DAILY dose.*

1. ***Initial IV methylprednisolone dose***

*If IV methylprednisolone was prescribed as well please enter the initial infusion dose.*

***At 6 months***

*The BSR guidelines recommend: “Disease assessment should occur monthly during remission induction and every 3 months during initial maintenance treatment and thereafter every 6 months and then annually (2b/B). Final consensus 100%. Tools assessing damage (VDI) should be used at baseline, 6 and 12 months (2b/B). Final consensus 100%.”*

*For the purposes of this audit the standard was relaxed to disease assessment at 6 months only*

1. ***BVAS score:*** *as per Q1****.***
2. ***Was the patient in remission at 6 months?.*** *Please answer based on whether formal indication in medical record of this status. Drug free remission is off steroids and not on any maintenance treatment.*
3. ***Assessment of disease activity and extent:*** *as per Q2****.***

4

**ANCA Vasculitis Audit Guidance Notes November 2015**

1. ***VDI (vasculitis damage index) at 6 months:*** *please enter if recorded, otherwise leave blank.*
2. ***Prednisolone dose at 6 months:*** *please enter the DAILY dose*

***Q11-13:*** *Please tick whether this patient received cyclophosphamide (Cyc), and/or rituximab or had initial remission induction with an alternative agent. Any question that is answered will then open a subsequent screen related to this drug*

***FOR PATIENTS RECEIVING CYCLOPHOSPHAMIDE BETWEEN 1 APRIL 2013 AND 31 DECEMBER***

***2014 FOR NEW DIAGNOSIS OR RELAPSE***

***Q 14-20: Date, dose and route of administration of cyclophosphamide, patient age, weight and creatinine.***

*These questions relate to the BSR guideline for cyclophosphamide ‘Treatment regimen’, which recommends “The standard dose [of pulsed IV cyclophosphamide] is 15 mg/kg, reduced for age and renal function (Table 1). The maximum IV CYC dose is 1500 mg.”*

*Table 1 Pulsed cyclophosphamide reductions for renal function and age*

| *Age (years)* | *Creatinine 150-300 µmol/l* | *Creatinine 300-500 µmol/l* |
| --- | --- | --- |
| *< 60* | *15mg/kg/pulse* | *12.5mg/kg/pulse* |
| *> 60 and < 70* | *12.5mg/kg/pulse* | *10 mg/kg/pulse* |
| *> 70* | *10 mg/kg/pulse* | *7.5mg/kg/pulse* |

***Q21: Was prophylactic medication co-administered (unless documented contraindication or not local policy)?***

*The BSR guideline recommends that “Mesna (2-mercaptoethane sulphonate sodium) should be considered for protection against urothelial toxicity in all patients receiving CYC, and especially in those receiving oral CYC”*

***Q22: FBC 7-10 days post 1^st^ dose of cyclophosphamide?***

*The BSR guideline recommends that “Patients on CYC should be monitored regularly for leucopaenia and the dose should be reduced if there is CYC induced leucopaenia/neutropaenia.*

*Between the first and second pulse, check the FBC on days 7 and 10 and the day of the pulse.*

*If the leucocyte nadir (minimum) is <3x10^9^/l and/or the neutrophil nadir is <1.5x10^9^/l even if the white blood cell (WBC) count has recovered to >4x10^9^/l and the neutrophil count is 2x10^9^/l on the day of the pulse, then reduce the dose of the next pulse by*

1. *Leucocyte nadir 1-2 x10^9^/l or neutrophil nadir 0.5-1.0 x10^9^/l: reduce CYC dose by 40%.*
2. *Leucocyte nadir 2-3 x10^9^/l or neutrophil nadir 1-1.5 x10^9^/l: reduce CYC dose by 20%. Before subsequent pulses check the FBC on the day of the pulse or the previous day.” For this audit, this standard was relaxed to one test between days 7 and 10.*

***Q23: Total cumulative dose of cyclophosphamide so far (including to the end of any current planned course)***

*The BSR guideline recommends that “Lifetime exposure to CYC should not exceed 25 g (3/C)” This is the cumulative dose of cyclophosphamide ever given to that patient.*

***Q24-26:*** *These relate to the BSR guideline section “Detection and prevention of potential adverse effects of immunosuppressive therapy”*

***Q27: At the most recent clinic visit were the following recorded: Urinalysis and BP?***

*This refers to the most recent clinic visit at the time the audit data is collected, for purposes of screening for haematuria post-cyclophosphamide, and cardiovascular assessment.*

***Q28-36:*** *These questions collect exactly the same information as Q 6-10 but only appear if the box is ticked indicating that CYC or RTX treatment has been given for RELAPSE.*

***Q37 Did this patient require admission to hospital with infection during cyclophosphamide therapy?*** *This question relates specifically to this course of cyclophosphamide therapy.*

*5*

**ANCA Vasculitis Audit Guidance Notes November *2015***

***Q38: Did this patient require admission to hospital with infection within 6 months of completing cyclophosphamide therapy?***

*Again, this question relates specifically to this course of cyclophosphamide therapy.*

***Q41-44:*** *These questions cover maintenance therapy once the cyclophosphamide therapy is finished.*

***FOR PATIENTS RECEIVING RITUXIMAB BETWEEN 1 APRIL 2013 AND 31 DECEMBER 2014 FOR***

***NEW DIAGNOSIS OR RELAPSE***

*If received 1^st^ course as induction therapy between 1 April 2013 and 31 December 2014*

***Q47-48:*** *These questions about use of cotrimoxazole or other pneumocystis jiroveci (PJP) prophylaxis and whether immunoglobulins were checked prior to treatment relate to safety checks prior to administering Rituximab. The summary of product characteristics for Rituximab states: Pneumocystis jiroveci pneumonia (PCP) prophylaxis is recommended for patients with granulomatosis with polyangiitis or microscopic polyangiitis during and following Rituximab treatment, as appropriate.*

***Q49: Which of the following criteria for Rituximab eligibility did the patient meet?***

*The NHS England commissioning policy in force at the time of this audit was published in April 2013, and the NICE Technology Appraisal in March 2014. Because these had slightly different eligibility criteria for the use of Rituximab, all of the potential eligibility criteria are included in this question – please tick all those that were relevant to this patient.*

*Please note that the* ***current*** *NHS England Commissioning Policy (which incorporates aspects of the NICE guidance) was published in January 2015, hence the audit time period ends in December 2014.* [*https://www.england.nhs.uk/commissioning/wp-content/uploads/sites/12/2015/01/a13-ritux-anca-vascul.pdf*](https://www.england.nhs.uk/commissioning/wp-content/uploads/sites/12/2015/01/a13-ritux-anca-vascul.pdf)

***Q51: Has any maintenance therapy been started?***

*Please include Rituximab here if it has been used as a maintenance (rather than remission induction only) agent.*

***Which agents have been used?***

***Q52-58:*** *These questions cover maintenance therapy used once the rituximab therapy is finished.*

***IF REMISSION INDUCTION WAS NOT WITH CYCLOPHOSPHAMIDE OR RITUXIMAB THERAPY: which agents have been used?***

*Complete if patient has received an alternative agent (i.e. not cyclophosphamide or rituximab) for a new*

*diagnosis of AAV between 1 April 2013 and 31 December 2014*

1. ***If remission induction was not with cyclophosphamide or Rituximab therapy, what was the reason?***

*The BSR guideline recommends “All patients with newly diagnosed AAV should be considered as having a potentially severe life or organ threatening disease and therefore be assessed for treatment with glucocorticoids and intravenous pulse cyclophosphamide (CYC) or rituximab (RTX)”*

*The rationale for this change in the guideline from previously is long term data from the NORAM study which compared methotrexate (MTX) with CYC in patients with no or minimal renal disease, and demonstrated that the median time to relapse was longer in CYC treated patients during the 18 month of the trial and the cumulative relapse-free survival was higher in the CYC group.*

***FOR ALL PATIENTS***

1. ***If known, what was the cause of death on the death certificate?***

*Please enter if available. It may be recorded in the patient notes, or as a letter or other document on the hospital electronic record system.*
